# Smoke-Free Policies in Multiunit Housing: Smoking Behavior and Reactions to Messaging Strategies in Support or in Opposition

**DOI:** 10.5888/pcd12.140479

**Published:** 2015-06-25

**Authors:** Carla J. Berg, Regine Haardörfer, Michael Windle, Madeleine Solomon, Michelle C. Kegler

**Affiliations:** Author Affiliations: Regine Haardörfer, Michael Windle, Madeleine Solomon, Michelle C. Kegler, Emory University Rollins School of Public Health, Atlanta, Georgia.

## Abstract

**Introduction:**

Given the high proportion of US adults living in multiunit housing (MUH) and the related risks of secondhand smoke, we examined correlates of having smoke-free MUH policies, level of support for such policies, and reactions to related messaging among a quota-based nonprobability sample of US adults.

**Methods:**

In 2013, 752 adult MUH residents were recruited through an online survey panel to complete a cross-sectional survey assessing tobacco use, personal smoke-free policies in homes and cars, smoke-free MUH policies, and reactions to messaging on smoke-free MUH policies. We sought sufficient representation of smokers, racial/ethnic minorities, and residents of the Southeast.

**Results:**

Overall, 56.3% had no smoke-free MUH policies and 16.2% had complete policies; 62.8% favored living in smoke-free MUH, and 28.9% said they would move if their building became smoke-free. Multivariate regression indicated that correlates of living in MUH with partial or no policies included younger age, less education, lower income, and current smoking (*P*’s ≤ .01); more restrictive smoke-free MUH policies were associated with lower cigarette consumption and recent quit attempts among current smokers (*P*’s < .05); and correlates of support for MUH policies included greater education, nonsmoker status, and having complete MUH policies (*P*’s < .05). Of 9 messages opposing smoke-free MUH policies, the most persuasive was “People have the right to smoke in their own homes”; the most persuasive message of 11 in support was “You have the right to breathe clean air in your home.”

**Conclusion:**

Smoke-free MUH policies may reduce smoking. Messaging in favor of smoke-free MUH policies was more persuasive than messaging opposing such policies, indicating the potential for using these approaches.

## Introduction

The home is a significant source of secondhand smoke exposure (SHSe) (1,2). Despite support for smoke-free public policies (3,4), research documenting support for policies in personal living areas is limited (5). Because smoke can penetrate building cracks and move through doorways and stairwells easily (6–8), people in multiunit housing (MUH) complexes are vulnerable to SHSe from neighboring units and common areas (9). One-third of American housing units are rented; 65% are in MUH complexes (10). Thus, MUH complexes are important settings for addressing the risks of SHSe. One study detected nicotine in 89% of nonsmoking homes among 49 low-income multiunit residences (9); another estimated that SHS infiltrated the homes of 27.6 to 28.9 million MUH residents with smoke-free home rules in the previous year (11). Those in affordable MUH are at higher risk of SHSe than those in other types of MUH and the general population (9). Women and people living below the poverty line are more likely to live in MUH (11). Additionally, children and the elderly are more likely to live in affordable MUH, and a substantial proportion of MUH residents have a chronic condition exacerbated by SHSe (eg, asthma) (9).

Research suggests that smokers living in MUH with smoking restrictions may be more likely to reduce or quit smoking, particularly indoors, resulting in less SHSe (12). Moreover, most MUH residents support a smoke-free MUH policy (13–16). However, much of this research has focused on certain cities (13,17,18) or states (13–16). One national survey of US adults living in MUH (19) reported that 29% lived in a smoke-free building and of those with personal smoke-free home policies, 56% supported a complete smoke-free MUH policy. Efforts to support or oppose tobacco control policies use arguments related to the impact of such policies on health, economic issues, prevention of youth tobacco use, individual rights, hospitality, and morality (20–22). However, to our knowledge, no research has examined the most persuasive messaging strategies that support or oppose MUH smoking policies or examined these issues in a nationwide sample.

This study drew from a socioecologic framework (23) and examined 1) sociodemographics in relation to level of smoke-free MUH policies, 2) sociodemographics and level of smoke-free MUH policies in relation to level of cigarette consumption and past year quit attempts among current smokers, and 3) sociodemographics, smoking status, and level of smoke-free MUH policies in relation to support for smoke-free MUH policies in a sample of US adults living in MUH with representation from key groups such as smokers and racial/ethnic minorities. We also examined the persuasiveness of messaging strategies in support of and in opposition to smoke-free MUH policies. A primary aim was to compare reactions to tobacco control policies in the southeastern United States (where tobacco control is lagging) with reactions in other regions. 

## Methods

### Procedures

This study was approved by Emory University’s institutional review board and involved a secondary analysis of cross-sectional survey data collected from participants in an online consumer panel, GMI (Global Market Insite, Inc), during 3 weeks (June 20–July 9, 2013). GMI has its own proprietary panel; recruitment techniques include Web advertising, public relations, partner-recruited panels, and alliances with heavily trafficked portals. GMI’s US panel is roughly 65% female; 50% has an annual income below $46,000, and its racial diversity is similar to that of the nation (roughly 75% white, 12% black). The average duration of panel membership is 9 to 12 months. GMI’s surveys are conducted via email and online; participants complete an average of 1.7 surveys per month.

Eligible participants lived in the United States, spoke English, and were aged 18 to 65 years. We used a group-targeted sampling quota approach to ensure that we had sufficient representation of individuals who 1) used a combustible tobacco product (ie, cigarettes, cigars, pipes) in the previous year (capped at 40%), 2) were from racial/ethnic minority populations (capped at 40%), and 3) resided in a southeastern state (ie, Alabama, Florida, Georgia, Kentucky, Mississippi, North Carolina, South Carolina, or Tennessee) (capped at 30%). Although not a probability-selected sampling approach (which is expensive), this relatively low-cost nonprobability sampling plan addresses our main research questions on reactions to tobacco control policies with sufficient representation among these key populations.

Participants were recruited through daily email invitations to GMI panelists. After panelists entered the survey, they were presented with the informed consent page. Those who consented were directed to screening questions to assess eligibility. If the quota for a particular subgroup was filled, panelists with those characteristics were no longer recruited. Participants were compensated with points that could be exchanged for items or gift cards in GMI’s system.

Data were managed by GMI and stored in a secure network. The research team had access to a portal that allowed them to monitor response rates from key demographic groups (eg, sex, age). The research team and GMI were able to make decisions about capping enrollment of subgroups, but it was not necessary. Once data collection was complete, the data set was made available to the research team.

### Participants

Overall, 5,429 participants began screening for study eligibility; 1,248 did not meet eligibility criteria, and 1,182 were ineligible because of full quotas. Of the 2,999 remaining, 252 discontinued before completing the eligibility screening, 243 were eligible but discontinued the survey, and the responses of 3 participants were eliminated by the survey company during their quality check process to ensure that no participant completed the survey more than once. The final study sample was 2,501 (response rate of 83.4%) and consisted of complete data (because the survey required an answer to each question before moving on to the next). As a result of quota-restricted sampling, 36.7% (n = 918) were current smokers (ie, smoked in previous 30 days), 31.6% (n = 791) were from racial/ethnic minority populations, and 26.7% (n = 669) resided in the Southeast. Our analyses focused on the 752 (30.1%) participants who indicated they resided in MUH (a “townhome/duplex” or “apartment/condominium/multiunit complex”).

### Measures

Our measures were based on a socioecologic framework (23), wherein spheres of influence affect one another on the individual and policy levels. On an individual level, we examined tobacco use behavior (level of tobacco use, recent quit attempts, and personal smoke-free policies), attitudes toward smoke-free MUH policies, and reactions to messaging strategies that support or oppose smoke-free MUH policies. On a policy level, we assessed smoke-free MUH policies in home communities. All measures were self-reported.

We assessed age, sex, race/ethnicity, education, household income, relationship status, number of people in the home, and number of children in the home (24).

We assessed previous 30-day use of cigarettes, electronic cigarettes, hookah, any cigar product, any smokeless tobacco use, and marijuana, using measures from the Centers for Disease Control and Prevention’s National Adult Tobacco Survey (24). Among current cigarette smokers, we assessed days smoked in the previous month, cigarettes per day, and number of previous-year quit attempts (24).

All participants were asked, “Which statement best describes the rules about smoking inside your home? Do not include decks, garages, or porches: Smoking is not allowed anywhere inside my home; Smoking is allowed in some places or at some times; or Smoking is allowed anywhere inside the home” and “Which statement best describes the rules about smoking inside your car? Smoking is not allowed anywhere inside my car; Smoking is allowed in my car sometimes; Smoking is allowed in my car; or I don’t own a car” (24).

Measures on experience with and attitudes about smoke in MUH were adapted from prior research (25) through development and review by an expert panel (including tobacco control practitioners, researchers in smoke-free policies, psychometricians, and statisticians). Participants living in MUH were asked, “Which statement best describes the landlord’s or property manager’s rules about smoking. Would you say the landlord or property manager: Has no rules about smoking; Allows smoking only in designated areas; or Doesn’t allow smoking anywhere.” Participants were also asked, “Can you sometimes smell smoke from another apartment or unit in your own unit?”; “Would you like to live in a smoke-free building where no one is allowed to smoke inside the building at all, including inside their own apartment or unit?”; and “Would you choose to move if your building became smoke-free?”

We also asked participants to rate the extent to which they perceived messaging strategies that support or oppose MUH smoke-free policies to be persuasive on a scale of 1 (not at all persuasive) to 9 (extremely persuasive). The messages were adapted from prior work (26,27), framed on 6 issues (health, youth tobacco use prevention, economic impact, individual rights and responsibility, morality and religion, and hospitality), and reviewed by our expert panel.

### Data analyses

Participant characteristics, smoking and smoking-related policies, and reactions to messaging were summarized using descriptive statistics. To investigate bivariate relationships while accounting for the quota sampling of minorities, smokers, and those living in southeastern states, we conducted multivariate regression analyses and binary, multinomial, and ordinal logistic regression analyses to examine differences among those with no smoke-free MUH policies, partial (ie, some smoke-free areas) policies, and complete (smoke-free in all indoor areas) policies. Subsequently, we conducted a series of multivariate regression models including other covariates. We conducted 1) an ordinal regression examining sociodemographics in relation to level of smoke-free MUH policies, 2) an ordinary least squares regression examining sociodemographics and level of MUH policies in relation to number of days of smoking in the previous 30 days among smokers, 3) a binary logistic regression model examining sociodemographics and level of smoke-free MUH policies in relation to past-year quit attempts among smokers; and 4) 2 binary logistic regression models examining sociodemographics, smoking-related factors, and level of smoke-free MUH policies in relation to support for smoke-free MUH policies (ie, would like them implemented or would move if implemented). Because level of mandated smoke-free MUH policies would have obvious implications for personal smoke-free homes, personal smoke-free policies were not included as a potential correlate in the regressions. For each regression, we used backward stepwise entry of the correlates of interest. We then compared reported persuasiveness of each message between MUH residents who are smokers versus nonsmokers, controlling for minority status and residence (Southeast versus other). All statistics were conducted using SPSS 21.0 (IBM Corporation); α was set at .05.

## Results

### Correlates of smoke-free MUH policies

In this sample, 423 (56.3%) participants had no smoke-free MUH policies; 207 (27.5%) had partial policies; and 122 (16.2%) had complete policies (Table 1). Living in MUH with partial or complete smoke-free policies was more common among younger people (*P* < .001), those with higher incomes (*P* = .003 for income of $50,000–$75,000; *P* = .03 for income ≥$75,000), and those who reported not smoking cigarettes in the previous 30 days (*P* = .005). Ordinal regression using backward stepwise regression including sociodemographics and smoking status indicated that correlates of living in MUH with partial or no smoke-free policies were younger (parameter estimate = −0.02; 95% confidence interval [CI], −0.03 to −0.01; *P* < .001), had less education (parameter estimate = −0.68; 95% CI, −1.11 to −0.26; *P* = .002), had lower income (parameter estimate = −0.77; 95% CI, −1.36 to −0.18; *P* = .01), and were current smokers (parameter estimate = 0.39; 95% CI, 0.09–0.68; *P* = .01; Nagelkerke R^2^ = .067). Additionally, those with partial or no smoke-free MUH policies were less likely to have private smoke-free policies in their homes than those with complete policies (*P* < .001 and *P* = .03, respectively) (Table 2).

**Table 1:** Self-Reported Participant Characteristics and Bivariate Analyses Examining Differences Among MUH Residents by Level of Smoke-Free MUH Policy^a^

Variable	MUH Residents (n = 752)	Level of Smoke-Free MUH Policy			
		No Policy (n = 423)	Partial Policy^b^ (n = 207)	Complete Policy^c^ (n = 122)	*P* Value^d^
**Demographics**					
**Age, mean (SD), y**	39.4 (14.5)	41.3 (14.1)	36.7 (14.6)	37.8 (14.5)	<.001
**Sex**					
Male	388 (51.6)	229 (54.1)	101 (48.8)	58 (47.5)	.12
Female	364 (48.4)	194 (45.9)	106 (51.2)	64 (52.5)	
**Race**					
White	431 (57.3)	238 (56.3)	120 (58.0)	73 (59.8)	—
Black	185 (24.6)	114 (27.0)	44 (21.3)	27 (22.1)	.55
Other	136 (18.1)	71 (16.8)	43 (20.8)	22 (18.0)	.14
**Education**					
≤High school diploma	174 (23.1)	117 (27.7)	32 (15.5)	25 (20.5)	—
Some college	316 (42.0)	174 (41.1)	94 (45.4)	48 (39.3)	.004
≥Bachelor’s degree	262 (34.8)	132 (31.2)	81 (39.1)	49 (40.2)	.50
**Income**					
<$25,000	278 (37.0)	142 (33.6)	91 (44.0)	45 (36.9)	—
$25,000 to <$50,000	243 (32.3)	153 (36.2)	58 (28.0)	32 (26.2)	.21
$50,000 to <$75,000	178 (23.7)	107 (25.3)	40 (19.3)	31 (25.4)	.003
≥$75,000	53 (7.0)	21 (5.0)	18 (8.7)	14 (11.5)	.03
**Employment status**					
Full-time	288 (38.3)	172 (40.7)	73 (35.3)	43 (35.2)	—
Part-time	131 (17.4)	63 (14.9)	40 (19.3)	28 (23.0)	.31
Other	333 (44.3)	188 (44.4)	94 (45.4)	51 (41.8)	.04
**Relationship status**					
Married or has partner	332 (44.1)	187 (44.2)	92 (44.4)	53 (43.4)	.95
Other	420 (55.9)	236 (55.8)	115 (55.6)	69 (56.6)	
**Household size and composition**					
Mean no. of people in home (SD)	2.4 (1.5)	2.5 (1.7)	2.3 (1.4)	2.3(1.2)	.28
Has children in home	206 (27.4)	125 (29.6)	52 (25.1)	29 (23.8)	.21
**Previous 30-Day Use of Smoking Products**					
Cigarettes	300 (39.9)	178 (42.1)	97 (46.9)	25 (20.5)	.005
E-cigarettes	55 (7.3)	29 (6.9)	25 (12.1)	1 (0.8)	.97
Hookah	29 (3.9)	12 (2.8)	15 (7.2)	2 (1.6)	.26
Any cigar product	104 (13.8)	60 (14.2)	32 (15.5)	12 (9.8)	.97
Any smokeless tobacco	32 (4.3)	10 (2.4)	20 (9.7)	2 (1.6)	.06
Marijuana	95 (12.6)	53 (12.5)	34 (16.4)	8 (6.6)	.95

Abbreviations: MUH, multiunit housing; SD, standard deviation.

a All values are number (percentage) unless otherwise indicated.

b Allows smoking only in designated areas.

c Does not allow smoking in any indoor areas.

d All *P* values were adjusted by controlling for smoking status, minority racial/ethnic category, and residence (southeastern United States vs other) with the outcome of level of smoke-free MUH policies.

**Table 2:** Experiences With MUH Smoke-Free Policies and Private Smoke-Free Policies Among MUH Residents (n = 752)^a^

Variables	All MUH Residents (n = 752)	Nonsmokers (n = 452)	Current Smokers (n = 300)	*P* Value^b^	Level of Smoke-Free MUH Policies				
					No Policy (n = 423)	Partial Policy^c^ (n = 207)	Complete Policy^d^ (n = 122)	*P* Value,^b^ No Policy vs Complete Policy	*P* Value,^b^ Partial Policy vs Complete Policy
**Smoking factors^e^**									
No. of days smoked in past month (SD)	—	—	—	—	9.7 (13.3)	9.8 (112.9)	2.6 (7.5)	<.001	.002
No. of cigarettes per day (SD)	—	—	—	—	10.3 (8.8)	9.9 (7.6)	4.8 (5.9)	.002	.009
Ready to quit in next month	—	—	—	—	16 (9.0)	16 (16.5)	8 (32.0)	.002	.10
Recent quit attempt	—	—	—	—	73 (41.0)	52 (53.6)	15 (60.0)	.06	.56
**Home smoking policies**									
Not allowed anywhere	478 (63.6)	1,339 (84.6)	440 (47.9)	<.001	232 (54.8)	140 (67.6)	106 (86.9)	<.001	.03
Allowed in some places or times	138 (18.4)	144 (9.1)	230 (25.1)		80 (18.9)	47 (22.7)	11 (9.0)		
Allowed	136 (18.1)	100 (6.3)	248 (27.0)		111 (26.2)	20 (9.7)	5 (4.1)		
**Car smoking policies**									
Not allowed anywhere	382 (50.8)	1264 (79.8)	262 (28.5)	<.001	202 (47.8)	96 (46.4)	84 (68.9)	.12	.09
Allowed some times	118 (15.7)	106 (6.7)	224 (24.4)		73 (17.3)	35 (16.9)	10 (8.2)		
Allowed	125 (16.6)	74 (4.7)	352 (38.3)		81 (19.1)	40 (19.3)	4 (3.3)		
I don’t own a car	127 (16.9)	139 (8.8)	80 (8.7)		67 (15.8)	36 (17.4)	24 (19.7)		
**Reactions to smoke-free MUH**									
Can smell smoke from another unit	293 (39.0)	181 (40.0)	112 (37.3)	.32	150 (35.5)	93 (44.9)	50 (41.0)	.42	.42
Would like to live in a smoke-free building	472 (62.8)	348 (77.0)	124 (41.3)	<.001	227 (53.7)	141 (68.1)	104 (85.2)	<.001	.047
Would move if MUH became smoke-free	217 (28.9)	93 (20.6)	124 (41.3)	<.001	130 (30.7)	61 (29.5)	–	.26	.54

Abbreviations: — , not applicable; MUH, multiunit housing; SD, standard deviation.

a All values are number (percentage) unless otherwise indicated.

b All *P* values were adjusted by controlling for smoking status, racial/ethnic minority category, and residence (southeastern United States vs other) with factors listed in each row as outcomes.

c Allows smoking only in designated areas.

d Does not allow smoking in any indoor areas.

e Among 918 current (previous 30 day) smokers overall and 300 smokers in MUH.

### Association of smoke-free MUH policies and smoking behaviors among current smokers

Among current cigarette smokers, living in MUH with partial or no smoke-free policies was associated with more days of smoking in the previous 30 days (*P* < .001 and *P* = .002, respectively) and more cigarettes per day (*P* = .002 and *P* = .009, respectively) (Table 2). In the regression, correlates of more days of smoking in the previous 30 days were older age (β = 0.07; 95% CI, 0.01–0.14; *P* = .02), not being married or living with a partner (β = 3.08; 95% CI, 1.22–4.94; *P* = .001), and partial or no smoke-free MUH policies (β = −2.64; 95% CI, −3.84 to −1.44; *P* < .001; adjusted R^2^ = .254). In the regression, correlates of past-year quit attempts were being from a racial/ethnic minority population other than black (odds ratio [OR] = 2.23; 95% CI, 1.08–4.61; *P* = .03), fewer days of smoking in the previous 30 days (OR = 0.97; 95% CI, 0.95–0.99; *P* = .03), and partial or complete smoke-free MUH policies (OR = 1.49; 95% CI, 1.02–2.19; *P* = .04; Nagelkerke R^2 ^= .087).

### Reactions to smoke-free MUH policies

Among MUH residents, 39.0% reported smelling smoke from another apartment or unit, 62.8% said they would like to live in a smoke-free MUH complex, and 28.9% said they would move if their building became smoke-free (Table 2). Current smokers were less likely than nonsmokers to say they would like to live in a smoke-free MUH complex and more likely to say they would move if such a policy were implemented (*P*’s <.001). Those with partial or no smoke-free MUH policies were less likely to report that they would like to live in a smoke-free MUH complex (*P* < .001 and *P* = .047, respectively). In the regression, correlates of support for MUH policies were greater education (OR = 1.28; 95% CI, 1.02–1.61, *P* = .03), nonsmoker status (OR = 0.93; CI, 0.92–0.94; *P* < .001), and having partial or complete smoke-free MUH policies (OR = 1.86; 95% CI, 1.44–2.40; *P* < .001; Nagelkerke R^2^ = .300). In the regression among MUH residents without a complete smoke-free MUH policy, correlates of reporting that they would move if smoke-free MUH policies were implemented included being white (OR = 2.35; 95% CI, 1.54–3.58, *P* < .001) and a current cigarette smoker (OR = 1.04; 95% CI, 1.03–1.06, *P* < .001; Nagelkerke R^2^ =.113).

### Reactions to messaging strategies

Smokers rated all messages opposing smoke-free MUH policies (except the one framed by morality and religion) as more persuasive than nonsmokers did (all *P* values <.001; Table 3). Smokers rated 9 of the 11 messages in support of MUH policies as less persuasive than nonsmokers did. The messages opposing or supporting smoke-free MUH policies framed on individual rights and responsibilities were reported to be the most persuasive.

**Table 3:** Persuasiveness of Messages Opposing or Supporting Smoke-Free MUH Policies Among MUH Resident Nonsmokers (n = 452) and Smokers (n = 300)

Issue	Message^a^	Nonsmokers, Mean Score (SD)^b^	Smokers, Mean Score (SD)^b^	*P* Value^c^
**Opposing**				
Health	We can accommodate both smokers and nonsmokers with common sense steps, like designating smoking areas and improving ventilation in apartment and condo complexes.	5.1 (2.6)	6.7 (2.1)	<.001
Youth tobacco use prevention	People with children are not forced to live in buildings where smoking is allowed. If they want to avoid smoke, they can find housing where it isn’t a problem.	4.0 (2.7)	5.7 (2.4)	<.001
Economic impact	Regulating smoke-free policies in apartment and condo complexes will cause them to close or lose revenue, which could negatively impact property maintenance and value.	3.5 (2.4)	5.0 (2.2)	<.001
Individual rights and responsibility	People have the right to smoke in their own home.	5.8 (2.5)	7.2 (2.0)	<.001
	Property owners, not the government, should decide whether to permit smoking in their properties. This decision is for them, not the government.	5.2 (2.8)	6.9 (2.1)	<.001
	People are not forced to live in buildings that allow smoking or to spend time in community spaces in multiunit complexes. If they want to avoid smoking, they should avoid places where smoking occurs in their buildings.	4.1 (2.3)	6.1 (2.2)	<.001
Morality and religion	Ensuring that we are accepting of smokers in our home community is a testament to God.	2.55 (2.2)	3.65 (2.6)	.17
Hospitality	Ensuring that smokers are comfortable in their own homes is respectful and reflects good manners.	4.7 (2.4)	6.0 (2.4)	<.001
**Supporting**				
Health	Smoke-free home rules lead to reduced secondhand smoke exposure and reduced smoking.	6.8 (2.1)	5.7 (2.3)	<.001
	Cigarettes are a major cause of residential fires and related deaths.	5.8 (2.2)	4.95 (2.3)	<.001
Youth tobacco use prevention	Youth who live in places that allow smoking in the home are more likely to become smokers.	6.0 (2.4)	5.4 (2.5)	<.001
Economic impact	New York realtors have reported that smokers’ residences are harder to sell than nonsmokers’ residences.	6.5 (2.3)	5.4 (2.4)	<.001
	Maine’s Sanford Housing Authority found that the cost of renovating smokers’ units ranged from $1,070–$1,670 versus $550 for a nonsmoking unit.	6.5 (2.4)	5.6 (2.3)	<.001
	Some insurance companies offer discounts on fire, life, liability, and property insurance to multiunit housing complexes that have adopted smoke-free policies.	6.5 (2.3)	6.0 (2.0)	<.001
Individual rights and responsibility	You have the right to breathe clean air in your home.	7.8 (1.8)	7.7 (1.7)	.42
	Your loved ones have the right to breathe smoke-free air in your home.	7.2 (2.4)	6.5 (2.6)	<.001
	Landlords and homeowners associations should be able to ban smoking in their properties.	6.8 (2.5)	6.1 (2.5)	.003
Morality and religion	Ensuring that we and our neighbors have clean air to breathe in our homes is a testament to God.	4.3 (2.9)	3.8 (2.6)	.11
Hospitality	Ensuring that everyone has clean air to breathe in their homes is respectful and reflects good manners.	6.9 (2.1)	6.3 (2.3)	<.001

Abbreviations: MUH, multiunit housing; SD, standard deviation.

a Messages were adapted from prior work (26,27).

b On a scale of 1 (not at all persuasive) to 9 (extremely persuasive).

c All *P* values were adjusted by controlling for racial/ethnic minority category and residence (southeastern United States versus other) with reactions to each message as outcomes.

## Discussion

Our findings indicated that people with complete smoke-free MUH policies engaged in less use of various tobacco products (ie, cigarettes, cigar products, electronic cigarettes, smokeless tobacco) and that, among current cigarette users, more restrictive smoke-free MUH policies were associated with less frequent smoking and greater likelihood of having made a quit attempt in the previous year. These findings parallel what is known about individually implemented smoke-free home policies (28,29) and prior literature on smoke-free MUH (12). Our results also align with what the socioecologic framework would suggest (23). The policy in one’s community (ie, whether smoking is allowed in one’s building) affects one’s tobacco use. As such, in addition to decreasing the negative health effects of SHSe among nonsmokers and children in MUH (30), such policies could lead to detectable decreases in the prevalence of smoking in the United States, especially since one-fifth of US adults live in MUH (10). 

People living in MUH with less restrictive smoke-free policies were younger and had less education and lower incomes. Widespread adoption of these policies could promote better health among populations vulnerable to tobacco use, such as those from lower socioeconomic backgrounds (31).

Among MUH residents, most said they would like to live in a smoke-free MUH complex; only a quarter said they would move if their building became smoke-free. Those most supportive of smoke-free policies were more likely to be nonsmokers, white, and from lower educational and socioeconomic levels. These findings are similar to prior findings indicating higher support among nonsmokers, racial/ethnic minorities, and those with less education (13–15,19).

Our study also showed that smokers in MUH reported that messages opposing smoke-free MUH policies were more persuasive and messages supporting such policies were less persuasive than nonsmokers reported them to be. However, the difference in ratings of persuasiveness between smokers and nonsmokers was not significant for 4 messages supporting smoke-free MUH policies. The messages rated as most persuasive both in opposition to and in support of smoke-free MUH policies were related to individual rights and responsibilities, and messages based on morality or religion were rated as least effective. On average, messages in support of smoke-free MUH policies were rated as more persuasive than those in opposition, which highlights the utility of such messages.

Our findings have important implications. Public health practitioners may promote support for smoke-free MUH policies by using messages framed on individual rights and responsibilities and proactively combat messages in opposition to such policies. Despite the fact that our sample is not representative of the US population, our findings suggest that most people would prefer a smoke-free complex and a minority would strongly oppose one. Thus, these policies may be appealing to MUH residents and managers.

This study has several limitations. The quota-based sample was drawn from a consumer panel population that may not represent the general US adult population. In addition, our restricted, quota-based sampling to obtain a high representation of people from racial/ethnic minority populations, recent tobacco users, and residents of the Southeast further limits the generalizability of these findings but was of value in feasibly addressing the research questions of interest. Estimates obtained with our data could be biased due to several factors, such as unmeasured variables associated with differential participation in the survey or differential participation by region of the country. Nevertheless, the quota-based sampling design enabled us to capture data with sufficient variation in factors (eg, racial/ethnic minorities, recent tobacco users) that were important to our research questions. The response rate for this study also implies some response bias; however, previous online research yielded much lower response rates (29%–32%) among the general US population (32). Also, the cross-sectional study design and the self-reported assessments limit the extent to which we can make causal attributions or account for bias. The use of stepwise regression analytic models is also subject to numerous difficulties (eg, biased coefficients that need shrinkage, collinearity of predictors, biased R^2^ values). Another limitation is that we did not allow participants to report “don’t know” for the question on level of smoke-free policies in their MUH complexes; doing so might have produced important information. Moreover, we did not account for level of public smoke-free policies, because we did not have complete information on the smoke-free policies in participants’ communities. These policies may have had an impact on the factors of interest, particularly smoking-related outcomes.

Broad adoption of smoke-free MUH policies could have a significant impact on health risks associated with SHSe, smoking prevalence, and cessation behaviors in the United States, given the large proportion of Americans living in MUH. In our study, most participants reported supporting such policies, with only a quarter reporting strong opposition. Messaging strategies in support of smoke-free MUH policies were more persuasive than those in opposition; messages framed on individual rights and responsibilities were most compelling. Policy makers and public health professionals can use these findings to develop messages that will garner support for the adoption of smoke-free MUH policies.
